# Di- and Tetrairon(III) μ-Oxido Complexes of an N3S-Donor Ligand: Catalyst Precursors for Alkene Oxidations

**DOI:** 10.3389/fchem.2019.00097

**Published:** 2019-03-01

**Authors:** Biswanath Das, Afnan Al-Hunaiti, Brenda N. Sánchez-Eguía, Erica Zeglio, Serhiy Demeshko, Sebastian Dechert, Steffen Braunger, Matti Haukka, Timo Repo, Ivan Castillo, Ebbe Nordlander

**Affiliations:** ^1^Chemical Physics, Department of Chemistry, Lund University, Lund, Sweden; ^2^Laboratory of Inorganic Chemistry, Department of Chemistry, University of Helsinki, Helsinki, Finland; ^3^Instituto de Química, Universidad Nacional Autónoma de México, Mexico, Mexico; ^4^Institute for Inorganic Chemistry, Georg-August-Universität Göttingen, Göttingen, Germany; ^5^Department of Chemistry, University of Jyväskylä, Jyväskylä, Finland

**Keywords:** Fe-S interaction, oxidation, homogeneous catalysis, thioether, iron-oxo complex

## Abstract

The new di- and tetranuclear Fe(III) μ-oxido complexes [Fe_4_(μ-O)_4_(PTEBIA)_4_](CF_3_SO_3_)_4_(CH_3_CN)_2_] (**1a**), [Fe_2_(μ-O)Cl_2_(PTEBIA)_2_](CF_3_SO_3_)_2_ (**1b**), and [Fe_2_(μ-O)(HCOO)_2_(PTEBIA)_2_](ClO_4_)_2_ (MeOH) (**2**) were prepared from the sulfur-containing ligand (2-((2,4-dimethylphenyl)thio)-N,N-bis ((1-methyl-benzimidazol-2-yl)methyl)ethanamine (PTEBIA). The tetrairon complex **1a** features four μ-oxido bridges, while in dinuclear **1b**, the sulfur moiety of the ligand occupies one of the six coordination sites of each Fe(III) ion with a long Fe-S distance of 2.814(6) Å. In **2**, two Fe(III) centers are bridged by one oxido and two formate units, the latter likely formed by methanol oxidation. Complexes **1a** and **1b** show broad sulfur-to-iron charge transfer bands around 400–430 nm at room temperature, consistent with mononuclear structures featuring Fe-S interactions. In contrast, acetonitrile solutions of **2** display a sulfur-to-iron charge transfer band only at low temperature (228 K) upon addition of H_2_O_2_/CH_3_COOH, with an absorption maximum at 410 nm. Homogeneous oxidative catalytic activity was observed for **1a** and **1b** using H_2_O_2_ as oxidant, but with low product selectivity. High valent iron-oxo intermediates could not be detected by UV-vis spectroscopy or ESI mass spectrometry. Rather, evidence suggest preferential ligand oxidation, in line with the relatively low selectivity and catalytic activity observed in the reactions.

## Introduction

The interaction between iron and sulfur in metalloproteins and in biomimetic molecular systems has attracted increased attention in recent years (Beinert et al., 1997; Ohnishi, 1998; Rao and Holm, 2004; Ballmann et al., 2008a; Meyer, 2008; Lill, 2009). Among these, the majority of the Fe/S structural motifs are dominated by clusters where the iron center is in distorted tetrahedral environments (Beinert et al., 1997; Meyer, 2008); nonetheless, there are examples of molecular systems where the Fe centers display diverse coordination geometries (Ballmann et al., 2008b; Fuchs et al., 2010). In some typical molecular systems, the metal center has also been found to be involved in secondary bonding interactions with ether-O and thioether-S units (Ballmann et al., 2008b). Although in all these examples sulfur, being a soft donor, displays preference for low valent Fe(II), there are molecular systems where the sulfur atom has been found to be bonded to high valent (III and IV) iron centers with distorted octahedral geometry (Harrop and Mascharak, 2004; McDonald et al., 2010; Widger et al., 2014). Overall, the Fe-S interaction in various molecular systems having iron in different formal oxidation states is an interesting field of research.

On the other hand, the selective and environmentally benign oxidation of hydrocarbons using affordable and efficient catalysts is another important area of modern synthetic chemistry. In this regard, bio-inspired iron chemistry has received increasing attention in recent decades due to the natural iron abundance in the earth crust, and the highly selective catalytic hydrocarbon oxidations of iron-containing oxygenases such as cytochrome P450 (Hasemann et al., 1995), soluble methane monooxygenase (sMMO) (Tinberg and Lippard, 2011), and Rieske dioxygenases (Wackett, 2002). Among the (catalytic) properties of these iron-based enzymes, the selective activation of C-H bonds under mild conditions is one of the most striking aspects. Many mono- and diiron complexes with various multidentate N-based ligands have been investigated in order to mimic the structures as well as functions of non-heme iron enzymes, and to potentially develop sophisticated oxidation catalysts (Costas et al., 2000, 2004; Sun et al., 2011; Lindhorst et al., 2015; Gamba et al., 2017). Such studies indicate that fine tuning of the coordination environment of the iron centers plays a crucial role in the catalytic activity, which includes O_2_ binding, followed by electron-transfer from Fe to O_2_ to afford either iron–superoxo (Fe^III^-${\text{O}}_{2}^{.-}$), iron–peroxo (Fe^III^-${\text{O}}_{2}^{2-}$), or iron–oxo (Fe^IV/V^ = O) species after initial O-O bond cleavage (Kim et al., 1997; Hazell et al., 2002; Rohde et al., 2003; Ye and Neese, 2011; De Visser et al., 2013; Wang et al., 2013a; Mitra et al., 2014; Nam, 2015).

A number of dinuclear Fe(III)-μ-oxido complexes relevant to the aforementioned enzymes from both structural and functional perspectives, have been studied as oxidation catalysts (Romakh et al., 2007; Visvaganesan et al., 2009; Wang et al., 2013a). Depending on the specific ligand environment, catalytic oxidation reactions using such dinuclear μ-oxido Fe(III) complexes can proceed via a radical intermediate or a high valent metal oxo species, or a combination of both, and the nature of these intermediates/active oxidants exerts a profound influence on the product distribution (Costas et al., 2000, 2004). The experimental evidence in all cases indicate that there is a strong influence of the ligand system on the catalytic activity, as well as the choice of oxidant (i.e., ultimate oxygen donor), which can also affect the product distribution (Costas et al., 2000, 2004). In spite of the extensive oxidative catalytic activity studies of these dinuclear Fe(III)-μ-oxido complexes, to the best of our knowledge there are very few well-characterized complexes that have been used as oxidation catalysts where sulfur occupies one of the coordination sites (McQuilken and Goldberg, 2012). The easily oxidizable nature of sulfur argues against its use in these types of systems (Widger et al., 2014), despite the fact that studying small molecular catalysts with sulfur-containing ligands can be very useful in modeling key metal-sulfur interactions that play a significant role in non-heme enzymes. Studies on sulfur oxygenation in a number of biomimetic non-heme iron(III)-thiolate complexes indicate that a long Fe-S bond distance makes the sulfur unit susceptible to attack by O_2_ in a reaction where iron maintains the +3 oxidation state (McQuilken and Goldberg, 2012). The active site of the non-heme iron enzyme cysteine dioxygenase (McCoy et al., 2006) is believed to pass through a Fe(III)-superoxo intermediate. DFT and QM/MM computational studies predict that the formation of the superoxo intermediate is followed by formation of an energetically favorable cyclic four-membered Fe-O-O-S ring structure that undergoes O-O bond cleavage to form a Fe(IV)(Oxo)-sulfinate analog. This metal oxo unit transfers the second oxygen atom to generate the cysteine-sulfinic acid product (Kumar et al., 2011; McQuilken and Goldberg, 2012).

Goldberg and coworkers have reported Fe(II) complexes of interesting pentadentate ligand systems incorporating one sulfur donor in efforts to model the reactivity of the active site of cysteine dioxygenase. While a pentadentate ligand with one thiolate donor is oxidized to the corresponding sulfoxide upon reaction with dioxygen, an analogous thioether ligand permits formation of a non-heme Fe(IV)oxo species that can perform oxygen atom transfer (Widger et al., 2014). In this context, it is worth mentioning that one of the most realistic biomimetic model systems (both in terms of structure and reactivity) for cysteine dioxygenase, [Tp^Me,Ph^Fe^II^CysOEt] (Tp^Me,Ph^ = hydridotris(3-phenyl-5-methylpyrazol-1-yl)borate), was reported by Limberg and coworkers (Sallmann et al., 2012), who used isotope (^16^O/^18^O) experiments to confirm that the treatment with dioxygen mainly leads to cysteine dioxygenase activity, i.e., deoxygenation of the bound cysteine ethyl ester.

**Figure 1:**
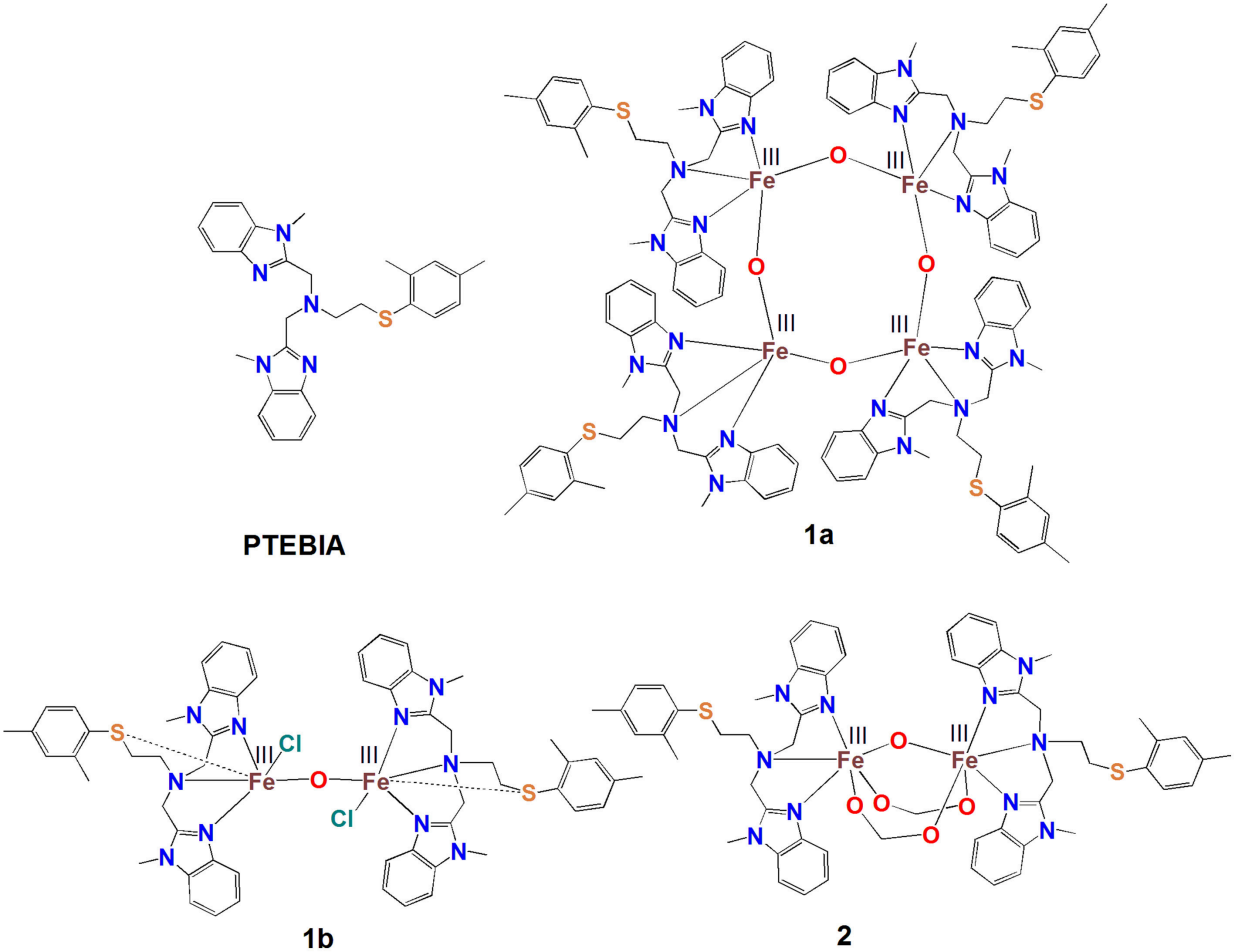
Schematic depiction of the structures of the PTEBIA ligand and complexes **1a**, **1b**, and **2**.

Here, we describe new di- and tetranuclear Fe(III)-μ-oxido complexes with the thioether-containing PTEBIA ligand (Castillo et al., 2012), *viz* [Fe_4_(μ-O)_4_(PTEBIA)_4_](CF_3_SO_3_)_4_(CH_3_CN)_2_] (**1a**), and [Fe_2_(μ-O)Cl_2_(PTEBIA)_2_](CF_3_SO_3_)_2_ (**1b**) (Figure 1). The Fe-S interaction in these complexes and their efficiency as homogeneous oxidation catalyst precursors will be discussed. The UV-vis and mass spectrometric investigations of plausible active species in solution are also presented. In order to gain a better understanding of the spectroscopic features of the complexes, specifically regarding the Fe(III)-S interaction, we have also synthesized the dinuclear complex [Fe_2_(μ-O)(μ-HCOO)_2_(PTEBIA)_2_](ClO_4_)_2_(MeOH) (**2**) that features additional bridging formate ligands.

## Results and Discussion

### Synthesis and Characterization of Complexes

**Figure 2:**
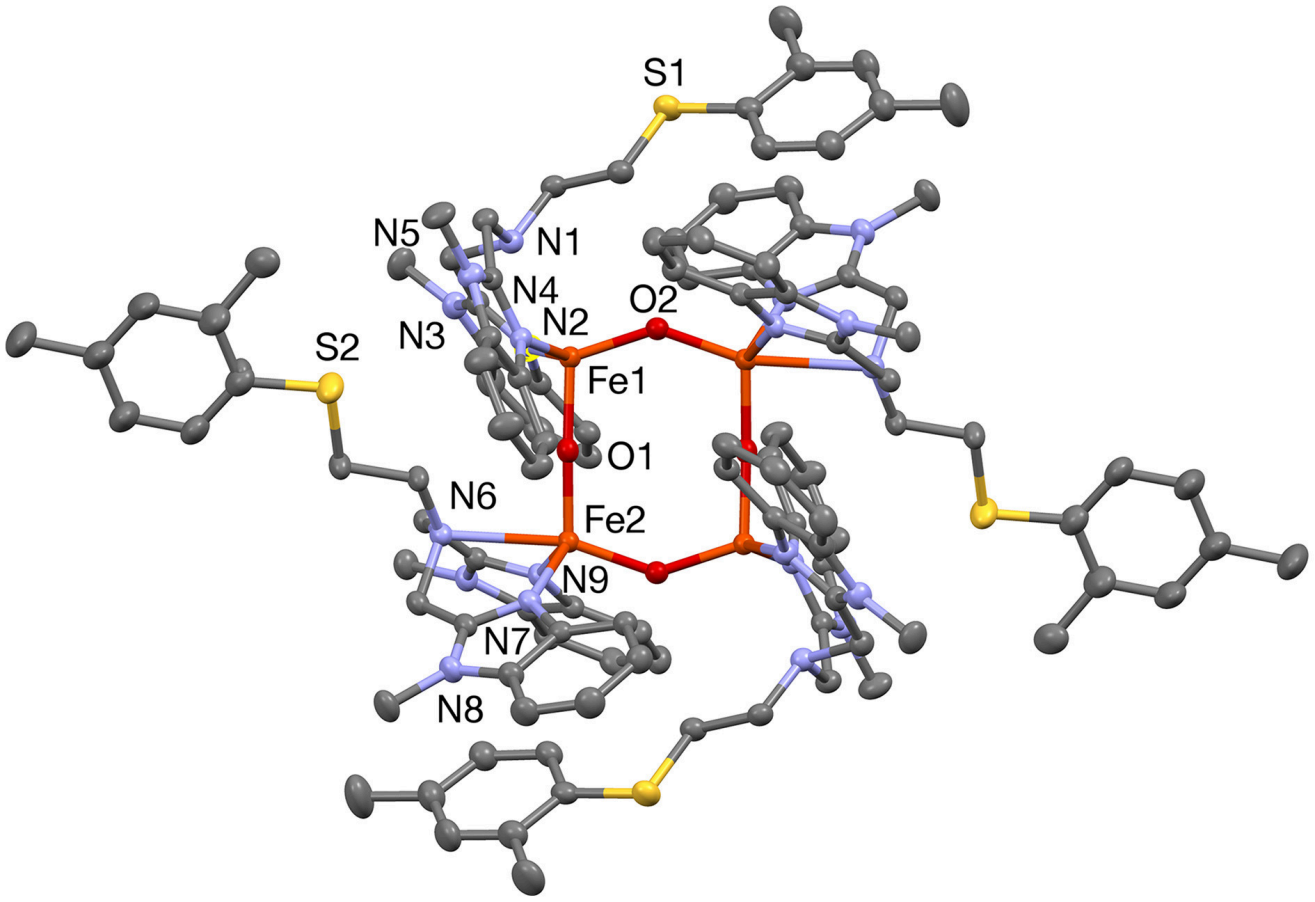
A plot (30% probability thermal ellipsoids) of the molecular structure of the cation of **1a**, [Fe_4_(μ-O)_4_(PTEBIA)_4_]^4+^ (hydrogen atoms and disorder omitted for clarity). Symmetry transformation used to generate equivalent atoms: (') 1/2–*x*, 3/2–*y*, 1–*z*.

The PTEBIA ligand was prepared following the procedure reported by Castillo and coworkers (Castillo et al., 2012). Addition of 1 equivalent of Fe(II)(OTf)_2_ (OTf = triflate, CF_3_${\text{SO}}_{3}^{-}$) to a tetrahydrofuran solution of PTEBIA leads to an immediate change of the color of the ligand solution to green. Refluxing of this green solution for 4 h, followed by crystallization by slow diffusion of diethylether into an acetonitrile solution of the product leads to yellow, needle shaped crystals of **1a** (Figure 2).

The solid-state structure of **1a** consists of a tetranuclear unit formed by four PTEBIA ligands, four bridging oxido ligands and four Fe(III) ions. To the best of our knowledge, this represents the first example of a tetrairon, tetra-oxido cluster with a sulfur-containing ligand. All the Fe(III) units have a distorted trigonal bipyramidal geometry, with N_3_O_2_ coordination environments. Selected bond lengths and bond angles for **1a** are listed in Tables 1, 2, respectively. There are two types of Fe-O-Fe bond angles and four different Fe-O bond distances present in the cluster. The angles are 142.36(15)° for Fe1-O2-Fe2′ and 171.64(18)° for Fe1-O1-Fe2 (Symmetry transformation used to generate equivalent atoms: (') 1/2–*x*, 3/2–*y*, 1–*z*). The corresponding bond lengths are 1.780(3) Å for Fe1-O2 and 1.778(6) Å for Fe2-O2,' and 1.811(3) Å for Fe1-O1, and 1.776(3) Å for Fe2-O1. The sulfur donor sites are > 5.2 Å away from the nearest Fe(III) unit, and thus there is no plausible intramolecular Fe(III)-S interaction.

**Table 1:** Selected bond lengths [Å] for **1a**.

**Atoms**	**Bond lengths**	**Atoms**	**Bond lengths**
Fe1–O2	1.780(3)	Fe2–N7	2.059(3)
Fe1–O1	1.798(3)	Fe2–N9	2.067(3)
Fe1–N2	2.076(3)	Fe1···Fe2'	3.3993(7)
Fe1–N4	2.081(3)	Fe1···Fe2	3.5646(9)
Fe2–O1	1.776(3)	Fe1···Fe1'	4.9297(8)
Fe2–O2'	1.811(3)	Fe2···Fe2'	4.9216(8)

*Symmetry transformation used to generate equivalent atoms: (') 1/2–x, 3/2–y, 1–z*.

**Table 2:** Selected bond angles [°] for **1a**.

**Atoms**	**Bond angles**	**Atoms**	**Bond angles**
O2–Fe1–O1	109.57(11)	O1–Fe2–N7	117.71(13)
O2–Fe1–N2	108.46(12)	O2'–Fe2–N7	97.80(12)
O1–Fe1–N2	98.24(12)	O1–Fe2–N9	114.80(13)
O2–Fe1–N4	113.86(13)	O2'–Fe2–N9	100.67(12)
O1–Fe1–N4	95.69(12)	N7–Fe2–N9	113.31(12)
N2–Fe1–N4	127.41(12)	Fe2–O1–Fe1	171.64(18)
O1–Fe2–O2'	109.46(11)	Fe1–O2–Fe2'	142.36(15)

*Symmetry transformation used to generate equivalent atoms: (') 1/2–x, 3/2–y, 1–z*.

**Figure 3:**
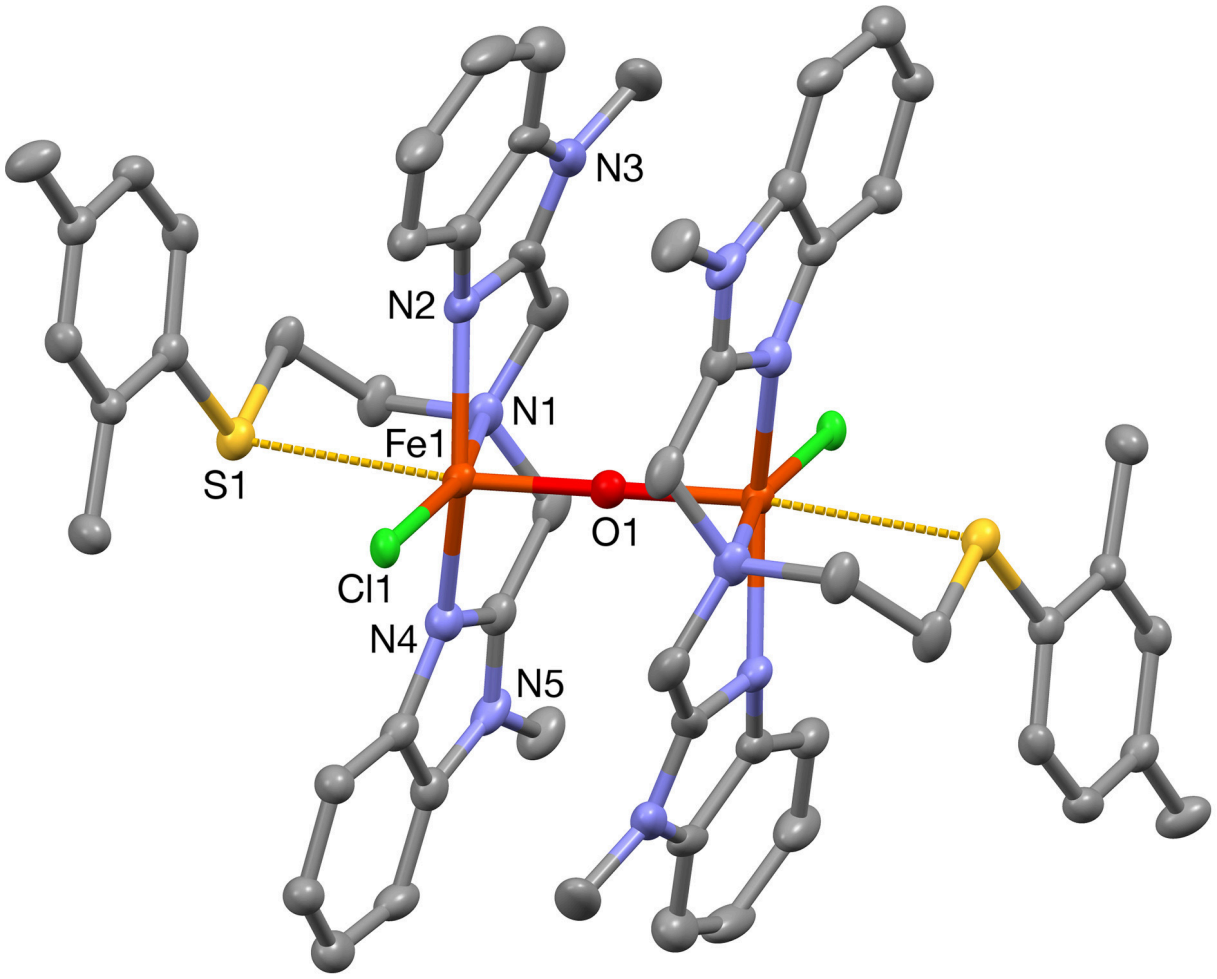
A plot (30% probability thermal ellipsoids) of the molecular structure of the cation of **1b**, [Fe_2_(μ-O)Cl_2_(PTEBIA)_2_]^2+^ (hydrogen atoms omitted for clarity). Only one of the two crystallographically independent molecules is shown. Symmetry transformations used to generate equivalent atoms: (') 1–*x*, 1–*y*, 1–*z*.

When PTEBIA contained a sub-stoichiometric amount of HCl from incomplete neutralization during the last synthetic step in the PTEBIA synthesis (as evidenced by the immediate precipitation of AgCl upon addition of AgNO_3_ to a chloroform solution of PTEBIA•*x*HCl) (Castillo et al., 2012), formation of **1a** was accompanied by brown crystals of **1b**; the latter complex was also prepared independently from FeCl_2_. Both complexes appear to be stable over a period of days in acetonitrile solution, but evaporation of the solvent results in oily products. Nonetheless, X-ray quality crystals of **1b** were isolated from cold acetonitrile solutions to acquire data at liquid N_2_ temperature. The molecular structure of the cation in [Fe_2_(μ-O)Cl_2_(PTEBIA)_2_](CF_3_SO_3_)_2_ (**1b**) is shown in Figure 3, and selected bond lengths and bond angles are collated in Tables 3, 4, respectively. In the solid state, **1b** is a μ-oxido diiron(III) complex, with Fe(III) centers in a slightly distorted octahedral N_3_SOCl coordination environment. The thioether moiety coordinates weakly with Fe(III)-S bond lengths of Fe-S bonds of 2.8364(17) and 2.8147(16) Å for the two crystallographically independent molecules found in the asymmetric unit (symmetry transformations used to generate equivalent atoms: (') 1–*x*, 1–*y*, 1–*z*; (”) 2–*x*, 1–*y*, –*z*). This Fe-S distance is long in comparison to Fe-S distances reported by Widger et al. for mononuclear Fe(II) complexes (~2.3 Å) with the thioether containing ligand N3Py^amide^SR (R = -(CH_2_)_2_CN) (Widger et al., 2014), but it is comparable to the long Cu(I/II)-S(thioether) distance observed for coordination of methionine in plastocyanin (2.9 Å) (Sahoo and Ray, 2007). The Fe(III) centers are oxido-bridged and are 3.5821(11) and 3.5809(11)Å (two crystallographically independent molecules) apart from each other with an Fe-O-Fe angle of 180°, which is in accordance with similar dinuclear Fe(III)-μ-oxido complexes reported by McKenzie and coworkers (Vad et al., 2012) and Wang et al. (2003).

**Table 3:** Selected bond lengths [Å] for **1b**.

**Atoms**	**Bond lengths**	**Atoms**	**Bond lengths**
Fe1–O1	1.7910(7)	Fe2–O2	1.7905(7)
Fe1–N2	2.085(4)	Fe2–N14	2.094(4)
Fe1–N4	2.095(5)	Fe2–N12	2.099(4)
Fe1–Cl1	2.2925(12)	Fe2–N11	2.306(4)
Fe1–N1	2.298(4)	Fe2–Cl2	2.3131(12)
O1–Fe1'	1.7910(7)	O2–Fe2”	1.7904(7)
Fe1–S1	2.8364(17)	Fe2–S2	2.8147(16)
Fe1···Fe1'	3.5821(11)	Fe2···Fe2'	3.5809(11)

*Symmetry transformations used to generate equivalent atoms: (') 1–x, 1–y, 1–z; (”) 2–x, 1–y, –z*.

**Table 4:** Selected bond angles [°] for **1b**.

**Atoms**	**Bond angles**	**Atoms**	**Bond angles**
O1–Fe1–N2	91.65(13)	O2–Fe2–N14	92.29(12)
O1–Fe1–N4	97.71(13)	O2–Fe2–N12	95.34(12)
N2–Fe1–N4	149.51(18)	N14–Fe2–N12	150.78(16)
O1–Fe1–Cl1	102.36(4)	O2–Fe2–N11	92.01(10)
N2–Fe1–Cl1	105.09(13)	N14–Fe2–N11	76.16(15)
N4–Fe1–Cl1	101.13(13)	N12–Fe2–N11	75.42(14)
O1–Fe1–N1	92.87(12)	O2–Fe2–Cl2	103.82(4)
N2–Fe1–N1	76.54(17)	N14–Fe2–Cl2	103.97(12)
N4–Fe1–N1	74.08(17)	N12–Fe2–Cl2	101.50(11)
Cl1–Fe1–N1	164.58(12)	N11–Fe2–Cl2	164.12(11)
Fe1–O1–Fe1'	180.00(4)	Fe2”–O2–Fe2	180.00(4)

*Symmetry transformations used to generate equivalent atoms: (') 1–x, 1–y, 1–z; (”) 2–x, 1–y, –z*.

**Figure 4:**
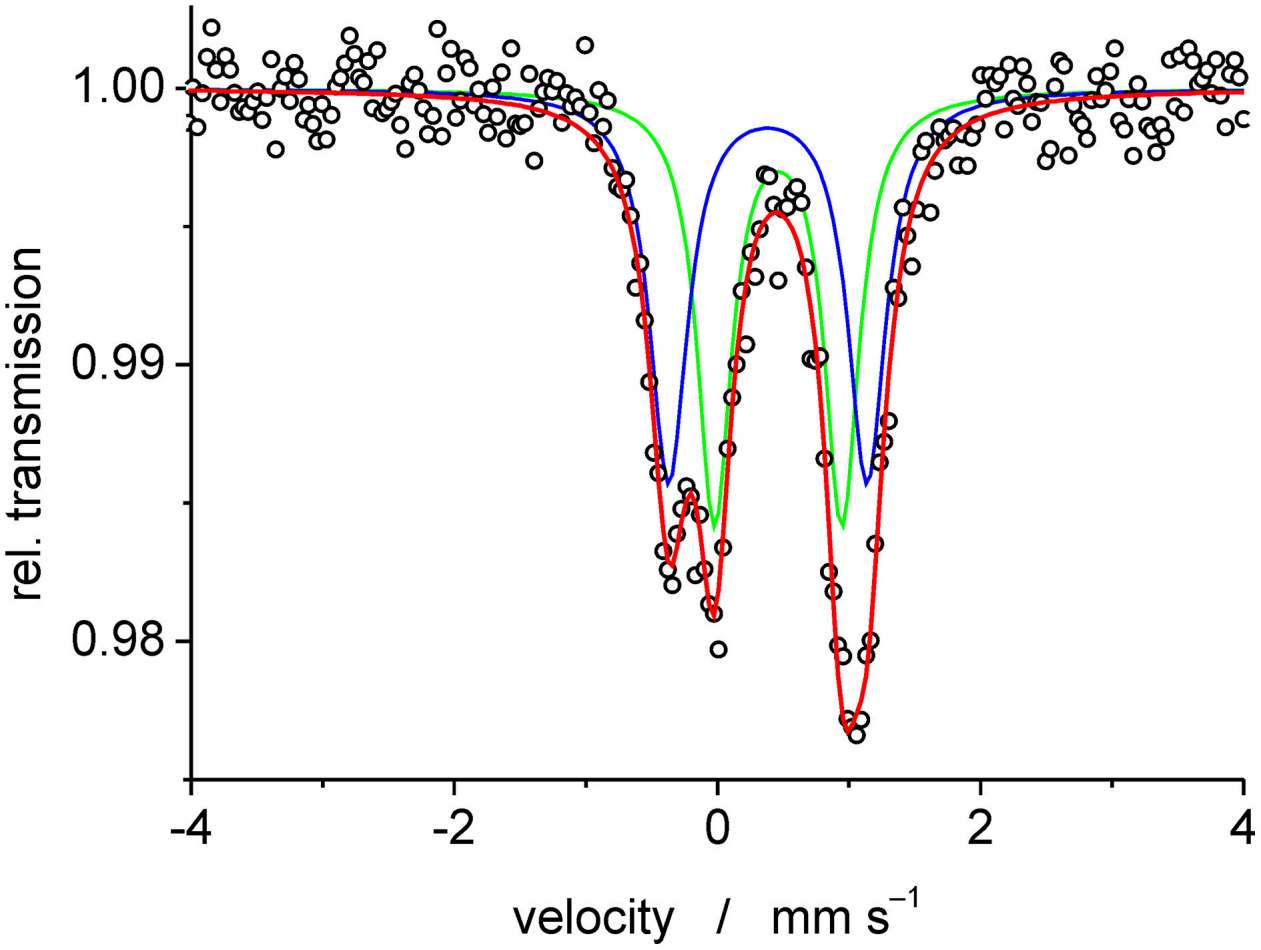
Experimental (open circles) and simulated (blue doublet*:* δ = 0.38 mm·s^−1^, Δ*E*_Q_ = 1.51 mm·s^−1^ and green doublet: δ = 0.46 mm·s^−1^, Δ*E*_Q_ = 0.97 mm·s^−1^) Mössbauer spectrum of complexes **1a** and **1b** recorded at 80 K.

Mass spectrometric measurements of **1a** gave rise to two major peaks corresponding to the mononuclear complex, i.e. at 673.7 amu for [Fe(PTEBIA)(CF_3_SO_3_)]^+^, and at 469.8 corresponding to protonated [HPTEBIA]^+^ (Figure S1). A peak arising from **1b** was also observed at 559.7 amu, assigned to [Fe(PTEBIA)(Cl)]^+^; no μ-oxido bridged species could be detected by either ESI or MALDI-TOF mass spectrometry. IR characterization revealed sharp resonances between 820 and 600 cm^−1^ assigned to asymmetric and symmetric Fe-O stretching modes of the Fe-O-Fe units in **1a** and **1b** (819, 749, 637 cm^−1^ in the former; 815, 780, 746, 633 cm^−1^ in the latter). Additional characterization of **1a** and **1b** was obtained by Mössbauer spectroscopy from 0.02 g of a batch of mixed crystals obtained from the reaction of Fe(OTf)_2_ with PTEBIA, revealing the presence of two high spin Fe(III) centers with different coordination environments in a 1:1 ratio of **1a** and **1b** (Figure 4). Since more ionic coordination environments with higher coordination numbers result in higher isomer shifts (δ), the subspectrum with δ = 0.46 mm·s^−1^ can be assigned to **1b**, whereas the subspectrum with δ = 0.38 corresponds to **1a**.

**Figure 5:**
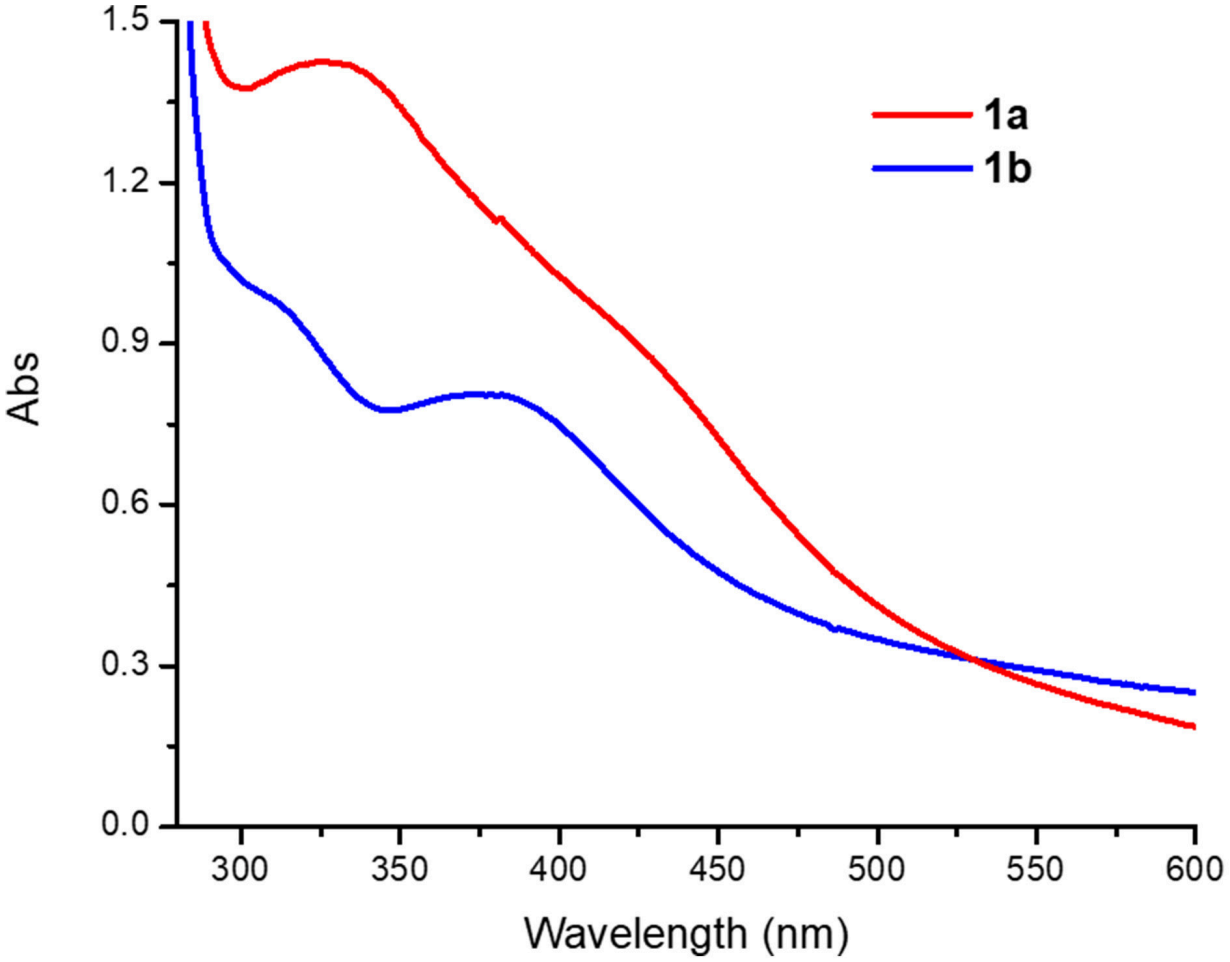
UV-Vis spectra of **1a** and **1b** in acetonitrile solution (0.4 mM).

Acetonitrile solutions of **1a** (0.4 mM) exhibit broad peaks with absorption maxima at 330 and 430 nm (ε = 3,600 and 2,280 M^−1^ cm^−1^, respectively), while **1b** features slightly narrower peaks with absorption maxima at 320 and 390 nm (ε = 2,400 and 1,990 M^−1^ cm^−1^, Figure 5). The peaks around 320–340 nm are in the so called “oxo dimer region” and are characteristic of Fe-O-Fe moieties, as has been observed for similar (μ-oxo)diiron(III) complexes (Reem et al., 1989; Kurtz, 1990; Do et al., 2012). The broad absorption bands around 390–430 nm can be assigned to weak sulfur to Fe(III) charge transfer bands (LMCT), similar to those observed in Cu(II) complexes with PTEBIA (Rodríguez Solano et al., 2011; Castillo et al., 2012).

Conversion of **1a** to **1b** was achieved in acetonitrile solution, as evidenced by UV-vis spectroscopy: addition of two equivalents of NBu_4_Cl as a source of chloride to 0.4 mM solutions of **1a** resulted in spectra that are virtually identical to those of the chlorido-containing **1b**, see Figure 5 and Figure S2.

**Figure 6:**
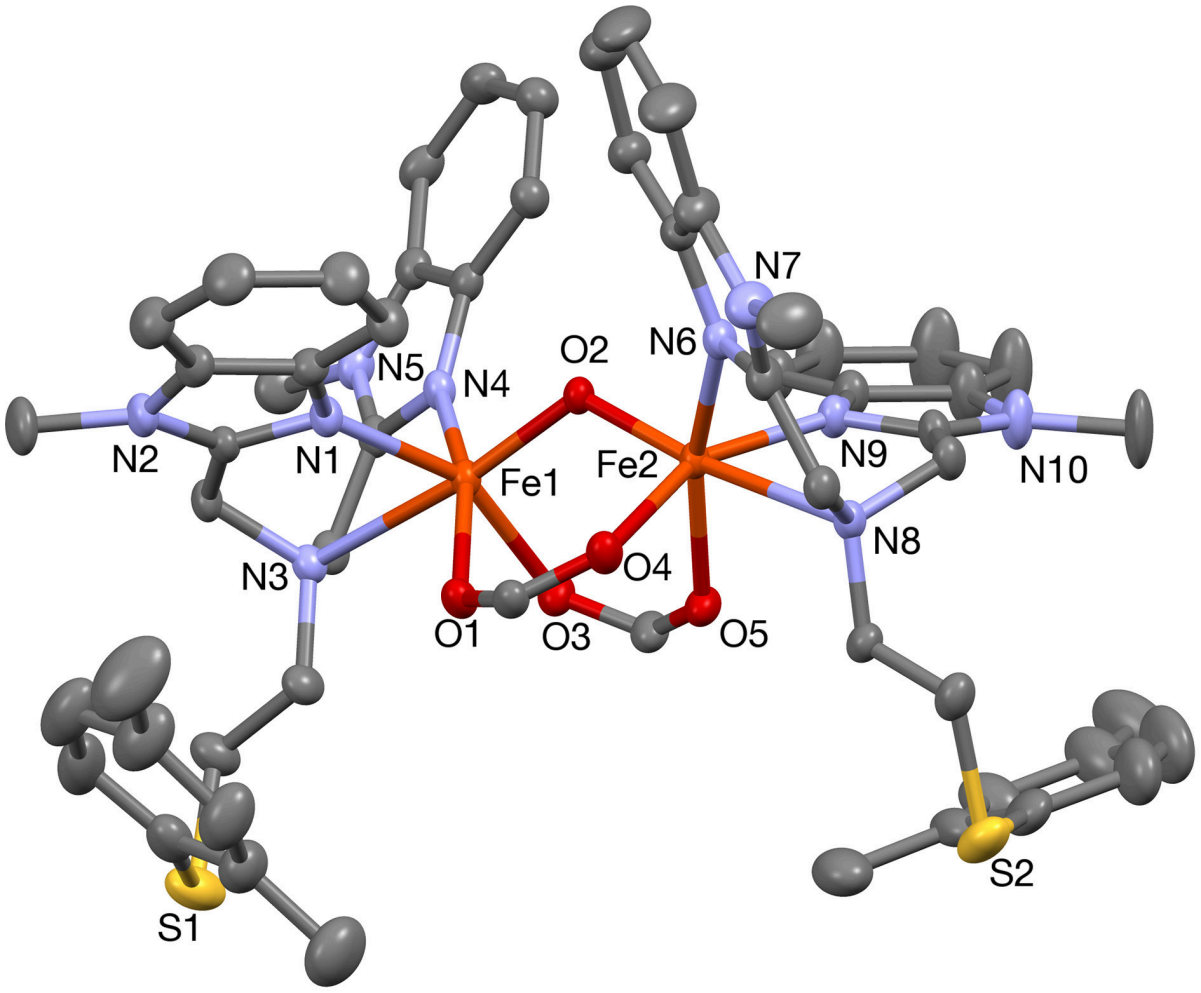
Molecular structure of [Fe_2_(μ-O)(HCOO)_2_(PTEBIA)_2_] (**2**). Thermal ellipsoids are plotted at 30% probability; all hydrogen atoms and perchlorate anions have been omitted for the sake of clarity.

To further probe the coordination mode of the PTEBIA ligand toward Fe(III) centers, equimolar amounts of Fe(II)(ClO_4_)_2_ and PTEBIA were heated to reflux in THF solution, followed by recrystallization from a methanolic solution by slow vapor diffusion of diethylether. Electrospray mass spectrometry on methanolic solutions of the green crystals obtained from the reaction show a major peak at 262.8 amu (see Figure S3), corresponding to the dicationic species [Fe(PTEBIA)]^2+^. The solid-state structure reveals that the complex consists of another μ-oxido diiron(III) species ([Fe_2_(μ-O)(HCOO)_2_(PTEBIA)_2_](ClO_4_)_2_(MeOH) (**2**), Figure 6), where both Fe(III) centers are in distorted octahedral environments with N_3_O_3_ donor sets. Selected bond distance and bond angles are collated in Tables 5, 6, respectively. The sulfur atoms are far apart from the Fe(III) centers (> 5.86 Å), ruling out any kind of direct interaction. The Fe(III) ions (Fe^…^Fe distance 3.121(9) Å) are coordinated by the N-donors of two PTEBIA ligands, as well as one bridging oxido and two formate ligands. The corresponding Fe-O-Fe angle is 121.046(1)°. The unexpected formate bridge is likely due to the aerobic oxidation of the methanol solvent in the presence of Fe(III) and PTEBIA, as has been observed by Que and co-workers in the related complex [Fe_2_(μ-O)(μ-HCOO)(TPA)_2_](ClO_4_)_3_ (TPA = tris(2-pyridylmethyl)amine) (Norman et al., 1998).

**Table 5:** Selected bond lengths [Å] for **2**.

**Atoms**	**Bond lengths**	**Atoms**	**Bond lengths**
Fe1–O1	2.094(4)	Fe2–O2	1.795(4)
Fe1–O2	1.792(4)	Fe2–O4	2.051(3)
Fe1–O3	2.039(3)	Fe2–O5	2.074(4)
Fe1–N1	2.081(3)	Fe2–N6	2.104(5)
Fe1–N3	2.378(5)	Fe2–N8	2.376(5)
Fe1–N4	2.100(5)	Fe2–N9	2.080(3)
Fe1–S1	5.861(2)	Fe2–S2	5.874(2)
Fe1···Fe2	3.122(2)	O2-S2	7.531(3)

**Table 6:** Selected bond angles [°] for **2**.

**Atoms**	**Bond angles**	**Atoms**	**Bond angles**
Fe2–Fe1–O1	74.0(1)	O1–Fe1–N1	81.7(1)
Fe2–Fe1–O2	29.5(1)	O1–Fe1–N3	90.6(1)
Fe2–Fe1–O3	80.5(1)	O1–Fe1–N4	163.5(1)
O1–Fe1–O2	97.7(1)	O2–Fe1–N1	103.0(1)
O1–Fe1–O3	86.1(1)	O2–Fe1–N3	171.3(1)
O2–Fe1–O3	99.5(1)	O2–Fe1–N4	98.6(1)
O2–Fe2–O4	98.3(1)	O3–Fe1–N1	155.6(2)
O2–Fe2–O5	97.8(1)	O3–Fe1–N3	83.7(1)
Fe2–Fe1–N1	115.7(1)	O3–Fe1–N4	88.9(1)
Fe2–Fe1–N3	158.6(1)	Fe1–Fe2–O2	29.4(1)
Fe2–Fe1–N4	120.6(1)	Fe1–Fe2–O4	80.1(1)

**Figure 7:**
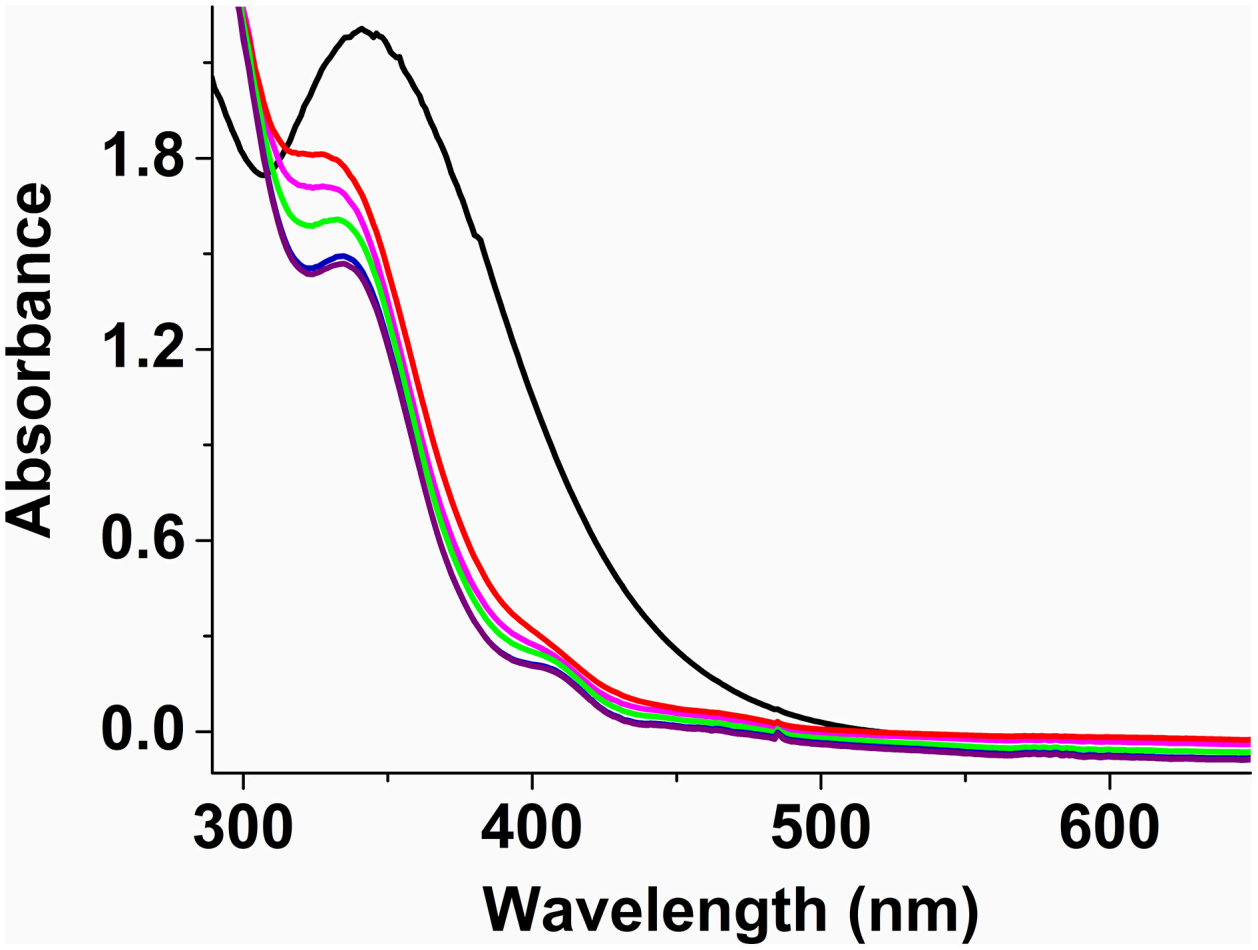
UV-Vis spectra of complex **2** in acetonitrile solution at 298 K (black), and after addition of H_2_O_2_/acetic acid mixture (1:1) at 228 K (purple), 238 K (blue), 248 K (green), 258 K (pink), and 268 K (red).

IR characterization of **2** shows characteristic asymmetric (ν_asym_) and symmetric (ν_sym_) C = O stretching bands as a broad peak at 1,614 cm^−1^ and two very sharp peaks for ν_sym_ at 1,490 and 1,453 cm^−1^, consistent with the presence of two bridging formates (Deacon and Phillips, 1980; Kurtz, 1990; Das et al., 2014a,b, 2018). In addition, the presence of a very sharp band at 1,080 cm^−1^ confirms the presence of perchlorate anions; the bands at 744 and 620 cm^−1^ correspond to the asymmetric and symmetric stretching modes of the Fe-O-Fe units (Kurtz, 1990; Norman et al., 1990). Acetonitrile solutions of **2** (0.5 mM) exhibit an absorption maximum at 342 nm (ε = 4,400 M^−1^ cm^−1^), characteristic of the Fe-O-Fe moiety (Kurtz, 1990; Norman et al., 1990; Do et al., 2012); unlike **1a** and **1b**, no lower energy bands were observed as the sulfur atom is not bound to the metal centers (Figure 7). Low temperature UV-vis experiments using a 1:1 (molar equivalent) of H_2_O_2_ (30 wt.% in H_2_O) and CH_3_COOH (> 99.7%) solution as oxidant and keeping overall complex:oxidant ratio at 1:2.5 reveal that the formate ligands of **2** may be protonated under these conditions, thus allowing the sulfur atom of PTEBIA to interact with the Fe(III) centers, as evidenced by the new absorption band at around 410 nm. This band is more prominent at low temperature (228 K) and appears only as a shoulder above 250 K (Figure 7) and was assigned to a S → Fe LMCT transition.

### Oxidation Catalysis

Jacobsen et al. (White et al., 2001) have effected efficient epoxidation of terminal long-chain alkenes using an iron complex based on the tetradentate N4-donor ligand mep (*N,N*'-dimethyl-*N,N*'-bis(2-pyridylmethyl)-ethane), H_2_O_2_ as oxidant and acetic acid as a promoter. These authors proposed that the active catalyst was the dinuclear ferric complex [Fe_2_(μ-O)(μ-OAc)(mep)_2_]^+^. Later studies by Fujita and Que indicated that the catalyst was rather the mononuclear complex [Fe(II)(mep)(solv)_2_]^2+^ (solv = solvent (NCMe)) (Fujita and Que, 2004). Similarly, Stack and coworkers (Dubois et al., 2003) used an Fe(III)-O-Fe(III) complex with aqua and nitrogen donor ligands, [Fe_2_(μ-O)(OH_2_)_2_(phen)_2_]^4+^ (phen = phenanthroline) as a catalyst/catalyst precursor for alkene epoxidation using peracetic acid as the oxidant. The combination of hydrogen peroxide with a suitable carboxylic acid (e.g., acetic acid) is believed to generate a peracid that in turn may generate a high valent metal oxo complex that functions as an active alkane/alkene oxidant (Fujita and Que, 2004), but it has also been suggested that the reaction of an Fe(II) complex with peracetic acid can lead to an Fe(III) κ^2^-peracetate complex with subsequent dissociation of the peracetate ligand to form a ferryl acyl radical species, i.e., an (OAc^.^)Fe(IV) = O species, that is an active oxidant (Wang et al., 2013b).

Palaniandavar and coworkers (Mayilmurugan et al., 2009) studied the use of Fe(III)_2_(μ-O) complexes, containing pentadentate Fe(III) ions chelated by tetradentate N_2_O_2_ salen-based ligands, as catalysts/precatalysts for the oxidation of alkanes and arenes using the peracid *meta*-chloroperbenzoic acid (*m*-CPBA) as the ultimate oxidant. With few exceptions, the obtained yields and alcohol/ketone ratios were relatively low, suggesting a mixture of metal-based and radical oxidation reactions. We have previously investigated the ability of Fe(III)_2_(μ-O) complexes with nitrogen and oxygen-based donor sets as active catalysts or precursors for the oxidation of alkanes and alkenes with hydrogen peroxide (Jarenmark et al., 2010; Das et al., 2015). A μ-oxo diiron(III) complex [{Fe(HIPCPMP)}_2_(μ-O)(Piv)]ClO_4_ (H_2_IPCPMP = 2-{*N*-isopropyl-*N*-[(2-pyridyl)methyl]aminomethyl}-6-{*N*(carboxymethyl)-*N*-[(2-pyridyl)methyl] aminomethyl}-4-methylphenol; Piv = Pivalate) showed moderate activity in cyclohexane oxidation, using H_2_O_2_ as the oxidant, and evidence suggests that both metal-based and radical mechanisms were involved in the process (Jarenmark et al., 2010). A greater contribution of the metal-based mechanism was found when the tetranuclear Fe_2_Li_2_ complex [${\text{Fe}}_{2}^{\text{III}}$O(LiDPCPMPP)_2_] [DPCPMPP = 3-[(3-{[bis(pyridin-2-ylmethyl)amino]methyl}-2-hydroxy-5-methylbenzyl)(pyridin-2-ylmethyl)amino]propanoate] was used as catalyst, based on the retention of configuration of the products observed in the oxidation of *cis*- or *trans*-1,2-dimethylcyclohexane (Das et al., 2015).

**Scheme 1:**
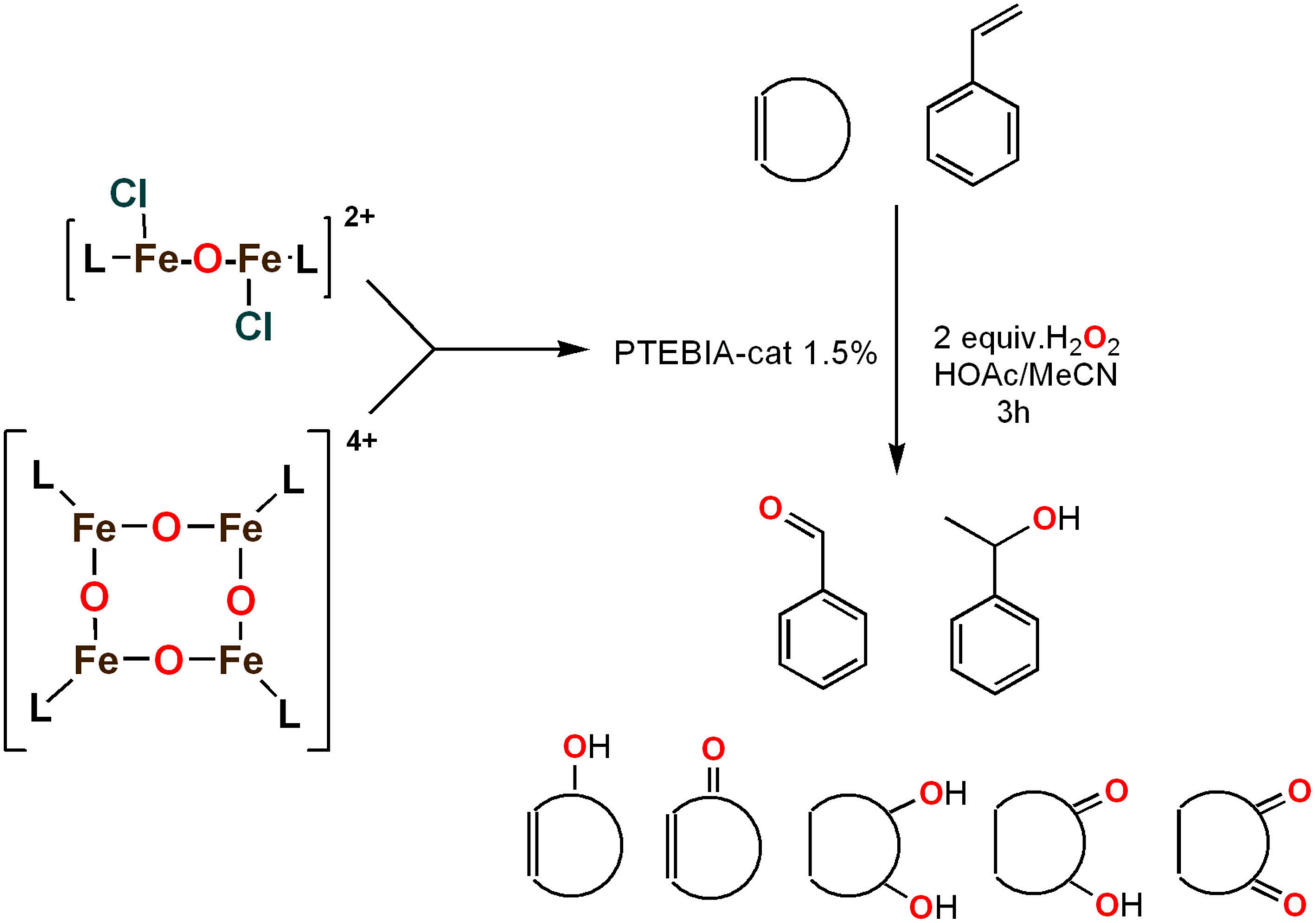
Schematic depiction of the catalytic oxidation reactions effected by complexes **1a**/**1b**; *cf*. text and Table 8.

To assess the effect of the mixed N_3_S donor set of PTEBIA on the activity and selectivity of iron complexes in the oxygenation of alkenes, we investigated the oxidation of styrene and a number of prototypical cyclic alkene substrates, viz. cyclohexene, 3-ethylcyclohexene, cyclooctene, under mild conditions (Scheme 1) using a bulk sample containing an approximate 1:1 mixture of **1a** and **1b** as catalyst precursor(s). The oxidation conditions were optimized by using cyclohexene as the model compound (Table 7). Based on the maximum reactivity and selectivity, acetonitrile was used as solvent with CH_3_COOH as additive and H_2_O_2_ as oxidant. These optimized conditions were applied in all oxidation experiments involving the different substrates. Mass spectrometry (ESI-MS) indicated that mixtures of **1a** and **1b** convert to dimeric [(Fe_2_)(OAc)_2_(μ-O)(PTEBIA)_2_(CF_3_SO_3_)]^+^ in acetonitrile solution, as evidenced by the peak detected at 1332.8 amu (Figure S4, Supplementary Material). Cyclohexene, cyclooctene and 3-ethylcyclohexene were oxidized in moderate conversions (29, 44, and 15%, Table 8) to the overoxidation products of the epoxides, namely diols and diketones. Although styrene oxidation (63% yield) afforded benzylmethanol (by epoxide ring opening), the formation of benzaldehyde as the major product indicates that the main active species is likely a transient radical (single electron oxidation). In comparison to the previously reported μ-oxido di-iron(III) complexes with N and O-based ligands [e.g., 2,6-bis(*N*-methylbenzimidazol-2-yl)pyridine (Wang et al., 2003), DPCPMPP (Das et al., 2015)], the catalytic efficiency (% of product formation and TON) of **1a** and **1b** is comparatively low (Romakh et al., 2007; Das et al., 2015).

**Table 7:** Optimization conditions using cyclohexene as a model substrate; substrate:catalyst:oxidant:AcOH (100:1.5:200:80), solvent 3 ml, 3 h reaction time.

**Entry**	**Temperature**	**Oxidant**	**Solvent**	**Conversion (%)**
1	10°C	H_2_O_2_	MeCN	7^a^
2	35°C	H_2_O_2_	MeCN	29^b^
3	50°C	H_2_O_2_	MeCN	31^c^
4	35°C	H_2_O_2_	MeCN	29^b^
5	35°C	O_2_	MeCN	2^a^
6	35°C	H_2_O_2_	H_2_O	8
7	35°C	H_2_O_2_	MeOH	13
8^*^	35°C	H_2_O_2_	MeCN	20^*^
9^**^	35°C	H_2_O_2_	MeCN	3^**^

a *^)^The main products are diol and 2-hexenol*.

b *^)^the main products are 2-hexen-1-ol*.

c *^)^poor selectivity*.

* *No acetic acid*.

** *No catalyst*.

**Table 8:** Reaction conditions: Substrate:catalyst:H_2_O_2_:AcOH (100:1.5: 200:80), MeCN as solvent 3 mL, temperature 35°C.

**Entry**	**Substrate**	**Time**	**Products(Yield [%])**	**TON**
1	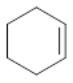	3	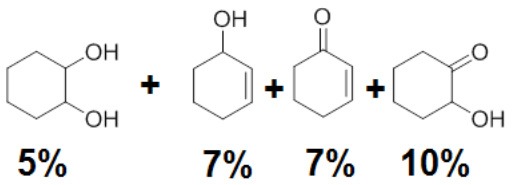	19
2	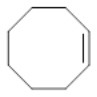	8	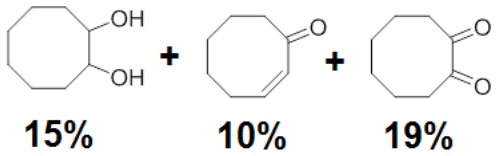	29
3	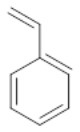	12	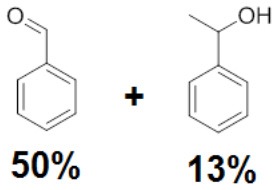	30
4	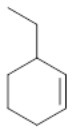	3	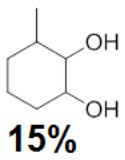	10

*Quantification by GC-MS experiments using 1,2-dichlorobenzene as internal standard. Time values are in hours*.

In addition to [(Fe_2_)(OAc)_2_(μ-O)(PTEBIA)_2_(CF_3_SO_3_)]^+^ detected by ESI MS, the main peaks in the mass spectra, and their isotopic patterns are consistent with species formulated as monomeric [Fe(HPTEBIA)(OH)]^+^, [Fe(HPTEBIA)(O)(OH)]^+^, [Fe(PTEBIA)(O)(CH_3_COO)]^+^, and the protonated form of the oxidized ligand [(HPTEBIA)(O)]^+^ (see Figures S4–S6, Supplementary Material), irrespective of whether **1a** and **1b** were analyzed separately or as a 1:1 mixture. This indicates that the active species present during turnover conditions appear to be identical regardless of the precursor. Preferential oxidation of the thioether moiety (generating the corresponding sulfoxide) over the metal center may be occurring, as reported by Goldberg and coworkers with the [Fe^II^(N_3_Py^amide^SR)](BF_4_)_2_ system in the presence of 5 equivalents of mCPBA (McQuilken and Goldberg, 2012). No evidence of the formation of Fe(IV) = O species was observed in analogous experiments with mCPBA or PhIO as oxo-transfer reagents. From the catalytic oxidation results induced by H_2_O_2_ and CH_3_COOH, we presume that catalysis with the current complex system does not involve heterolysis of the O-O bond to form a highly reactive Fe^(V)^O species, which should result in higher product selectivity (Prat et al., 2013). Instead, it may proceed *via* homolysis to give transient [LFe^(IV)^O] and hydroxyl radical (OH) intermediates, with participation of both species in the observed oxidations (Trettenhahn et al., 2006).

## Conclusion

We have synthesized and characterized a new tetrairon tetraoxo cluster (**1a**) with a sulfur-containing ligand. In the UV-vis spectroscopic experiments, a prominent sulfur to iron charge transfer band (390–430 nm) was observed at room temperature for **1a** and the corresponding dinuclear Fe(III)-O-Fe(III) complex **1b**, whereas for the related dinuclear dicarboxylate-bridged Fe(III)-O-Fe(III) complex **2** it was only visible at low temperature (228 K) in the presence of H_2_O_2_/CH_3_COOH (presumably after initial protonation/dissociation of the oxo and formate groups of **2**), and disappears at room temperature, likely due to loss of the Fe-S bond and/or sulfoxidation of PTEBIA. The catalytic efficiency of the μ-oxido iron(III) complex mixture of **1a** and **1b** for the oxidation of alkenes has been investigated, revealing that they act as moderate oxidation catalyst(s)/catalyst precursor(s). The reaction appears to proceed partially through a metal-centered process in tandem with free-radical oxidation by reactive oxygen species. Low temperature UV-vis spectroscopy and mass spectrometry were employed to gain insight into possible reactive intermediates/active oxidation catalysts, revealing that both H_2_O_2_/CH_3_COOH and PhIO preferably oxidize the thioether group of the ligand, in contrast with previous oxygenations with related copper-based PTEBIA systems, where the thioether functionality remains intact.

## Materials and Methods

The ligand PTEBIA was prepared following the procedure reported by Castillo et al. (2012). All reagents and solvents were of analytical or spectroscopic grade purchased from Sigma Aldrich, Fisher chemicals or VWR, and were used without further purification. Cyclohexene (≥ 99.0%) contained ~0.01% of 2,6-di-*tert*-butyl-4-methylphenol as stabilizer and was used as received. *Caution!* Even though no problems were encountered in this work, caution should always be taken while using high concentrations of hydrogen peroxide (H_2_O_2_), as well as metal perchlorates.

Infrared spectra were recorded in the 4,000–400 cm^−1^ range on a Nicolet Avatar 360 FTIR spectrometer, as KBr pellets. Mass spectra were obtained on a JEOL JMS-SX-102A mass spectrometer at an accelerating voltage of 10 kV with a nitrobenzyl alcohol matrix and Xenon atoms at 6 keV (FAB^+^), a JEOL JMS-AX505HA spectrometer (Electron Ionization), or a Bruker Daltonics Esquire 6000 spectrometer with ion trap (Electrospray). Elemental analyses were performed at the microanalytical facility of the Instituto de Química, UNAM, Mexico. Analytical achiral GC was performed on an Agilent 6850 GC with FID detector using an Agilent DB-WAX (30.0 m × 0.25 mm) column at mL/min He carrier gas flow. Chiral GC was performed on an Agilent 6850 GC with FID detector. The ^1^H NMR spectra were recorded with a Varian Gemini 200 apparatus or a Varian Mercury 300 MHz spectrometer.

Substrate conversions in catalytic experiments were determined by GC-MS. The GC-MS analyses were performed with an Agilent 6890 N Network GC system equipped with a DB-1MS column (30 m × 0.25 mm) and an Agilent 5973 Network MS detector. Calibration curves were obtained from commercial products purchased from Aldrich or TCI when available or from pure isolated products obtained from a catalytic reaction using a FID-detector GC with a HP-INOWAX column (30 m × 0.25 mm) (1,2-dichlorobenzene used as an internal standard). The concentrations of each organic product were calibrated relative to that of an internal standard (1,2-dichlorobenzene) with a known concentration.

### Syntheses

#### Synthesis of 1a

To 15 mL of a tetrahydrofuran solution of 0.13 g (0.26 mmol) of PTEBIA, 0.10 g (0.26 mmol) of Fe(II)(triflate)_2_ were added and the solution was refluxed for 4 h under vigorous stirring. Immediately after the addition of Fe(II)(triflate)_2_, the solution becomes turbid green, and on reflux it turns yellowish brown. The yellowish brown solution was filtered and the filtrate was collected in a 50 mL flask. Evaporation of the solvent under vacuum produces a tan oil. Overnight slow vapor diffusion of diethyl ether to a concentrated acetonitrile solution of the tan residue produces 0.12 g of **1a** [Fe_4_(μ-O)_4_(PTEBIA)_4_](CF_3_SO_3_)_4_(CH_3_CN)_2_ as yellow microcrystals (65%). UV-vis (CH_3_CN): λ_max_ = 335 nm (ε = 3,600 M^−1^ cm^−1^), 430 nm (ε~ 2,280 M^−1^ cm^−1^). ^57^Fe Mössbauer (80 K) δ = 0.55 mm/s; ΔEQ = 1.15 mm/s; ESI-MS in acetonitrile solution calculated for [Fe(PTEBIA)(CF_3_SO_3_)]^+^ (C_29_H_31_F_3_FeN_5_O_3_S_2_) (mononuclear species): 674.1; found 673.8. IR (KBr, cm^−1^): 2953, 2924, 2856, 1767, 1722, 1539, 1501, 1456, 1365, 1250, 1226, 1160, 1028, 967, 913, 819, 749, 636, 574, 517, 432. Anal. Calcd for C_116_H_124_F_12_Fe_4_N_20_O_16_S_4_: C, 50.44; H, 4.52; N, 10.14; S, 9.29; found: C, 50.19; H, 4.50; N, 9.88; S, 9.02.

#### Synthesis of 1b

The procedure is analogous to that for **1a**, employing 33 mg (0.26 mmol) of FeCl_2_. The brown solution was filtered and the filtrate was collected in a 50 mL flask. Evaporation of the solvent under vacuum produces a brown solid. Overnight slow vapor diffusion of diethyl ether to a concentrated acetonitrile solution of the brown solid affords 0.11 g of **1b** [Fe_2_(μ-O)Cl_2_(PTEBIA)_2_](CF_3_SO_3_)_2_ as brown microcystals [m.p. 195-197°C (dec)] (61%). UV-vis (CH_3_CN): λ_max_ = 320 nm (ε = 2,400 M^–1^ cm^–1^), 390 nm (ε ~ 1,990 M^–1^ cm^–1^). ^57^Fe Mössbauer (80 K) δ = 0.28 mm/s; ΔEQ = 1.31 mm/s; ESI- MS in acetonitrile solution calculated for [Fe(PTEBIA)(Cl)]^+^ (C_28_H_31_ClFeN_5_S) (mononuclear species): 560.1; found 559.7. IR (KBr, cm^–1^): 2935, 1736, 1597, 1491, 1452, 1426, 1378, 1256, 1223, 1151, 1100, 1054, 1029, 959, 932, 815, 780, 746, 700, 633, 572, 545, 516, 430. Anal. Calcd for C_58_H_62_Cl_2_F_6_Fe_2_N_10_O_7_S_4_: C, 48.51; H, 4.35; N, 9.75; S, 8.93; found: C, 48.32; H, 4.39; N, 9.00; S, 8.40.

#### Synthesis of 2

To a 20 mL tetrahydrofuran solution of 0.30 g (0.64 mmol) of PTEBIA, 0.16 g (0.64 mmol) of Fe(II)(ClO_4_)_2_ was added and the solution was refluxed for 3 h. The colorless solution of the ligand changes immediately to turbid green on addition of Fe(ClO_4_)_2_ and to yellowish green after 3 h of reflux. This yellowish green solution was filtered and the filtrate was dried under vacuum overnight in a 50 mL round bottom flask to get 0.64 g of 2 (74% yield). Slow vapor diffusion of diethyl ether for 4 days to the methanolic solution of 2 leads to needle-shaped yellowish green crystals of X-ray quality. UV-Vis (CH_3_CN): λ_max_ = 335 nm (ε = 4,400 M^–1^ cm^–1^). ESI-MS in acetonitrile solution calculated for [Fe(PTEBIA)]^2+^ (C_28_H_31_FeN_5_S)^2+^ (mononuclear species): 262.6; found 262.8. IR (KBr, cm^–1^): 3063, 3031, 2945, 2917, 1614, 1490, 1453, 1359, 1328, 1289, 1269, 1238, 1080, 928, 896, 865, 814, 795, 744, 695, 620, 545, 522, 489, 432. Anal. Calcd for 2: C, 51.38; H, 4.76; N, 10.33; S, 4.73; found: C, 50.99; H, 4.85; N, 10.45; S, 4.75.

### Oxidation Experiments

Each catalytic experiment was performed at least twice and the reported conversion is the average value. A general procedure for the oxidation experiments is as follows: magnetic stirring bar, catalyst complex (18 μmol), 2 mL of CH_3_CN, acetic acid (AcOH, 50 μL, 85 μmol), 230 μL of H_2_O_2_ (33% in water, 2.0 equivalents with respect to the substrate) and substrate (1 mmol) were placed in a Schlenk flask. The reaction mixture was stirred under argon at 35°C for the designated time. Sodium thiosulfate (ca. 400 mg, 2.5 mmol) was then added to the reaction mixture to quench further oxidation. 1,2-dicholorobenzene was added to the mixture followed by extraction with *n*-pentane and filtering through a silica gel column for analysis by GC-MS.

### X-Ray Structure Determination

Crystal data and details of the data collections are given in Table 9. X-ray data for complexes **1a** and **1b** were collected on a STOE IPDS II diffractometer (graphite monochromated Mo-Kα radiation, λ = 0.71073 Å) by use of ω scans at −140°C. The structures were solved by direct methods (SHELXS-2014) and refined on *F*^2^ using all reflections with SHELXL-2014 (Sheldrick, 2008). Non-hydrogen atoms were refined anisotropically. Hydrogen atoms were placed in calculated positions and assigned to an isotropic displacement parameter of 1.2 / 1.5 *U*_eq_(C). In each compound one of the two CF_3_${\text{SO}}_{3}^{-}$ ions was found to be disordered [occupancy factors: **1a** = 0.929(15)/0.071(15), **1b** = 0.784(4)/0.216(4)]. SAME restraints and EADP constraints were used to model the respective disorders. In **1a** one 2,4-dimethylphenyl moiety involving the carbon atoms C51(A/B/C) to C58(A/B/C) was found to be disordered about three positions A, B, and C along with acetonitrile (N21, C95, C96, belonging to B) and water (O31, belonging to C). After initial refinement the occupancy factors were set to 0.5 for A, 0.1 for B and 0.4 for C. RIGU, FLAT, SADI [*d*(1,3)_C(ar)···*C*(*Me*)_] and DFIX (*d*_C(ar)−C(Me)_ = 1.5 Å) restraints and EADP constraints were applied to model the disorder. The AFIX 66 instruction was applied for the carbon atoms of the ring. Furthermore, acetonitrile disordered about a 2-fold rotation axis and about two positions [occupancy factors: 0.277(7)/ 0.223(7)] was refined using RIGU restraints. Face-indexed absorption corrections were performed numerically with the program X-RED (Stoe & Cie and X-RED, 2005). The crystal of **2** was immersed in cryo-oil, mounted in a MiTeGen loop, and measured at a temperature of 170 K on a Rigaku Oxford Diffraction Supernova diffractometer using Mo Kα radiation. The *CrysAlisPro* (Rigaku Oxford Diffraction, 2013) software was used for cell refinement and data reduction. Empirical absorption correction based on equivalent reflections was [*CrysAlisPro* (Rigaku Oxford Diffraction, 2013)] was applied to the intensities before structure solutions. The structure was solved by charge flipping method using the *SUPERFLIP* (Palatinus and Chapuis, 2007) and the structure refinement was carried out using *SHELXL* (Sheldrick, 2015) program. The crystal of **2** contained solvent accessible voids but no satisfactory solvent model could be found. The contribution of the missing solvent to the calculated structure factors was taken into account by using the SQUEEZE routine of PLATON (Spek, 2009). The missing solvent was not taken into account in the unit cell content. Hydrogen atoms were positioned geometrically and constrained to ride on their parent atoms, with C-H = 0.95–0.99 Å, O-H = 0.84 Å, and U_iso_ = 1.2–1.5 U_eq_ (parent atom). The highest peak is located 1.40 Å from atom S1 and the deepest hole is located 0.59 Å from atom S1.

**Table 9:** Crystal data and refinement details for **1a**, **1b**, and **2**.

**Compound**	**1a**	**1b**	**2**
Empirical formula	C_126.40_H_139.60_F_12_Fe_4_N_25.20_O_16.80_S_8_	C_58_H_62_Cl_2_F_6_Fe_2_N_10_O_7_S_4_	C_59_H_68_Fe_2_N_10_O_14_S_2_Cl_2_
Formula weight	2988.50	1436.01	1387.95
Crystal size [mm3]	0.490 × 0.130 × 0.120	0.250 × 0.190 × 0.080	0.310 × 0.120 × 0.060
Crystal system	monoclinic	triclinic	monoclinic
Space group	*C*2/*c*	*P*−1	*C*2/*c*
*a* [Å]	27.6133(7)	12.1859(7)	41.374(5)
*b* [Å]	23.9349(9)	14.7606(9)	19.4162(6)
*c* [Å]	25.3002(6)	19.5751(11)	29.330(4)
α [°]	90	76.965(5)	90.00
β [°]	116.592(2)	88.005(5)	145.75(3)
γ [°]	90	66.307(4)	90.00
*V* [Å3]	14952.6(8)	3135.0(3)	13259(2)
*Z*	4	2	8
ρ [g/cm3]	1.328	1.521	1.391
*F*(000)	6,195	1,480	5,776
μ [mm^−1^]	0.573	0.759	0.650
*T*_min_ / *T*_max_	0.6874 / 0.9120	0.8369 / 0.9385	0.8242/0.9625
θ-Range [°]	1.245–25.670	1.549–25.680	2.821–30.097
*hkl*-range	−33 −28, ± 29, −30 −29	±14, ±17, −23 −21	−53 +30, 0.25, 0.38
Measured refl.	56,783	34,525	11,746
Unique refl. [*R*_int_]	14,101 [0.0808]	11,804 [0.0653]	15,192 [0.0384]
Observed refl. [*I* > 2σ(*I*)]	10,172	7,824	10,222
Data / restraints / param.	14,101 / 269 / 1,070	11,804 / 19 / 838	15,192/ 0/ 812
Goodness-of-fit (*F*^2^)	1.045	1.037	1.099
*R*1, *wR*2 (*I* > 2σ (*I*))	0.0654, 0.1632	0.0684, 0.1556	0.0625, 0.1795
*R*1, *wR*2 (all data)	0.0931, 0.1785	0.1108, 0.1748	0.0967, 0.1972
Resid. el. dens. [e/Å3]	−0.492 / 0.755	−0.411 / 1.196	−0.857/1.159

### Mössbauer Measurement

The Mössbauer spectrum was recorded at 80 K with a ^57^Co source in a Rh matrix, using an alternating constant-acceleration Wissel Mössbauer spectrometer operated in the transmission mode and equipped with a Janis closed-cycle helium cryostat or with a Mössbauer-Spectromag cryostat. Isomer shifts (cf. caption, Figure 3) are given relative to iron metal at ambient temperature. Experimental data were simulated using the *Mfit* software (developed by E. Bill, Max-Planck Institute for Chemical Energy Conversion, Mülheim/Ruhr, Germany, 2008).

## Data Availability

The raw data supporting the conclusions of this manuscript will be made available by the authors, without undue reservation, to any qualified researcher. Crystallographic data for the structures in this paper have been deposited with the Cambridge Crystallographic Data Center, CCDC, 12 Union Road, Cambridge CB21EZ, UK. Copies of the data can be obtained free of charge on quoting the depository number CCDC 1874712 (**1a**), 1874713 (**1b**) or 1874714 (**2**) (Fax:+44-1223-336-033; E-Mail: deposit@ccdc.cam.ac.uk, http://www.ccdc.cam.ac.uk).

## Author Contributions

BD performed all complex syntheses, performed low temperature studies in collaboration with BS-E, participated in the oxidation experiments, and contributed to the writing of the manuscript. AA-H performed all oxidation experiments and contributed to the writing of the manuscript. BS-E and EZ synthesized the PTEBIA ligand. SebD performed all Mössbauer measurements and analysis of the Mössbauer data. SerD, SB, and MH collected X-ray data and solved the crystal structures. TR supervised the oxidation studies and analyzed the results in collaboration with AA-H and BD, and supplied funding for the work. IC led and designed the study, contributed to the writing of the manuscript and supplied funding for the work. EN led and designed the study, contributed to the writing of the manuscript and supplied funding for the work.

### Conflict of Interest Statement

The authors declare that the research was conducted in the absence of any commercial or financial relationships that could be construed as a potential conflict of interest.
