# Supplementary material for: Di- and Tetrairon(III) μ-Oxido Complexes of an N3S-Donor Ligand: Catalyst Precursors for Alkene Oxidations
Source: Front Chem. 2019 Mar 1;7:97. doi: 10.3389/fchem.2019.00097 (PMC6405480; doi:10.3389/fchem.2019.00097)
Supplement: Supplementary file 1 [file Data_Sheet_1.docx]

Supplementary Material

Di- and Tetrairon(III) μ-oxido complexes of an N3S-donor ligand: catalyst precursors for alkene oxidations

Biswanath Das^1^, Afnan Al-Hunaiti^2^, Brenda N. Sánchez-Eguía^3^, Erica Zeglio^1^, Serhiy Demeshko^4^, Sebastian Dechert^4^, Steffen Braunger^4^, Matti Haukka^5^, Timo Repo^2^, Ivan Castillo^3*^, Ebbe Nordlander^1*^

*** Correspondence:** Ebbe Nordlander: [Ebbe.Nordlander@chemphys.lu.se](mailto:Ebbe.Nordlander@chemphys.lu.se)

# Supplementary Figures and Tables


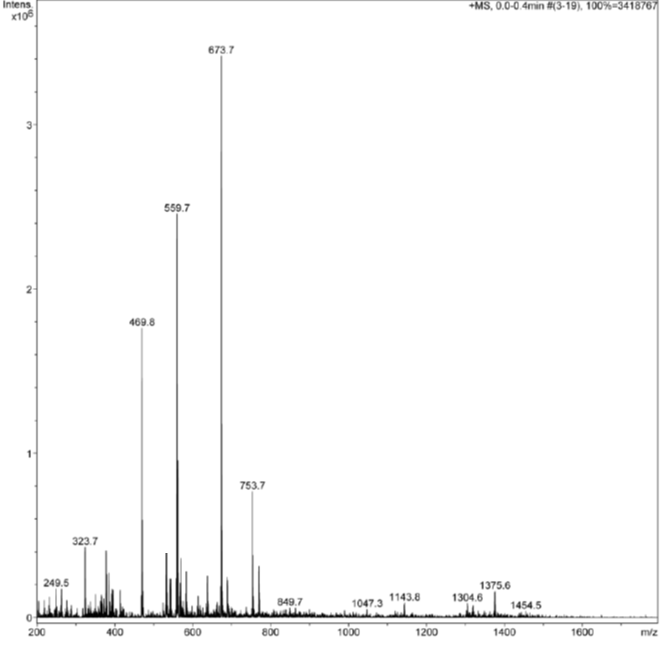


**Fig S1.** Mass spectrum (m/z 100 to 2000) of a mixture of complexes **1a** and **1b** in acetonitrile solution.

**Fig S2.** UV-Vis spectra of the conversion of **1a** to **1b** in acetonitrile solution upon addition of NH_4_Cl to the former (compare with Figure 4).

**Fig S3.** Mass spectrum (m/z 100 to 2000) of complex **2** in acetonitrile solution.

**Fig S4.** Mass spectrum (m/z 100 to 2000) of complexes **1a** and **1b** in acetonitrile solution in the presence of H_2_O_2_ (30 wt.% in H_2_O) and CH_3_COOH (> 99.7%, 1.049 g/mL) (1:1molar equivalent) solution as oxidant (overall complex:oxidant ratio 1:2.5).

**Fig S5.** Mass spectrum (m/z 414 to 506) of complexes **1a** and **1b** in acetonitrile solution in the presence of H_2_O_2_ (30 wt.% in H_2_O) and CH_3_COOH (> 99.7%, 1.049 g/mL, 1:1molar equivalent) solution as oxidant (overall complex:oxidant ratio 1:2.5).

**Fig S6.** Mass spectrum (m/z 530 to 640) of complexes **1a** and **1b** in acetonitrile solution in the presence of H_2_O_2_ (30 wt.% in H_2_O) and CH_3_COOH (> 99.7%, 1.049 g/mL, 1:1molar equivalent) solution as oxidant (overall complex:oxidant ratio 1:2.5). will be published under a Creative Commons CC-BY licence and permission must be obtained for use of copyrighted material from other sources (including re-published/adapted/modified/partial figures and images from the internet). It is the responsibility of the authors to acquire the licenses, to follow any citation instructions requested by third-party rights holders, and cover any supplementary charges.

**
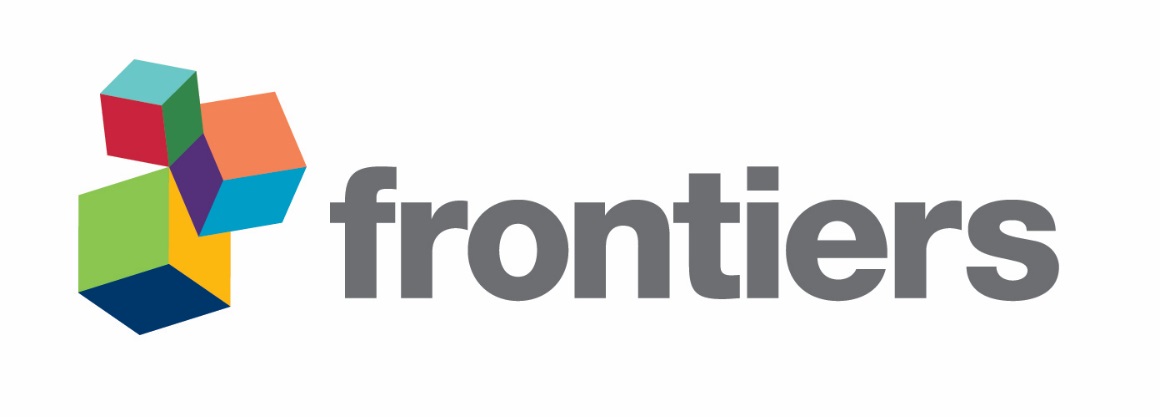
**

**Supplementary Figure 1.** The figure legends are required to have the same font as the main text, 12 point normal Times New Roman, single spaced. Please use a single paragraph for each legend and prepare the figures keeping in mind the PDF layout.
